# Lateral medullary syndrome resulting from atrial fibrillation due to rheumatic heart disease: A case report and literature review

**DOI:** 10.1002/ccr3.9124

**Published:** 2024-06-28

**Authors:** Sajina Shrestha, Suda Maharjan, Bandana Ghimire, Nischal Mainali, Kriti Gurung, Hritik Raj Yadav, Kriti Bhandari, Shumneva Shrestha, Anupam Halder, Kripa Rajak, Vikash Jaiswal

**Affiliations:** ^1^ KIST Medical College Lalitpur Nepal; ^2^ Kritipur Hospital, PHECT Kathmandu Nepal; ^3^ Nobel Medical College and Teaching Hospital Biratnagar Nepal; ^4^ Kathmandu Medical College Kathmandu Nepal; ^5^ Birat Medical College and Teaching Hospital Biratnagar Nepal; ^6^ M Abdur Rahim Medical College Dinajpur Bangladesh; ^7^ Nepal Medical College Kathmandu Nepal; ^8^ Maharajgunj Medical Campus, Institute of Medicine, Tribhuvan University Kathmandu Nepal; ^9^ Department of Internal medicine University of Pittsburgh Medical Centre Harrisburg USA; ^10^ Department of Research and Academic Affairs Larkin Community Hospital South Miami Florida USA

**Keywords:** atrial fibrillation, lateral medullary syndrome, rheumatic heart disease, stroke

## Abstract

Lateral medullary syndrome, resulting from cerebellar/brainstem infarction, can occur due to cardioembolic stroke from atrial fibrillation caused by rheumatic heart disease. This rare association highlights the importance of strict arrhythmia management, prophylactic anticoagulation, and timely diagnosis to prevent debilitating neurological outcomes.

## 1 INTRODUCTION

The lateral medullary syndrome (LMS) was first described by Gaspard Vieusseux in the early 1800s. In 1895 AD, Adolf Wallenberg provided a comprehensive description of LMS, alternatively known as Wallenberg syndrome, as a condition characterized by an occlusion in the posterior inferior cerebellar artery (PICA) or the vertebral artery (VA), resulting in an infraction in the medulla oblongata. More than 60,000 cases of LMS are diagnosed in the United States every year.^1^

Atrial fibrillation is a condition in which the heart rhythm is irregular and fast.^2^ Rheumatic heart disease (RHD) especially involving the mitral valve, is a major underlying factor for developing atrial fibrillation.^3^ The presence of atrial fibrillation increases the risk of thromboembolism and this dislodged embolus can cause the occlusion of PICA or VA, leading to Wallenberg syndrome.^2^, ^3^

Since there are few known cases relating LMS with AF due to RHD, this case report describes a case with both conditions and co‐relation between them.

We present a case of a 35‐year‐old female who has LMS due to RHD with atrial fibrillation and this case report adheres to the SCARE 2020 guidelines.^4^

## 2 CASE HISTORY OR EXAMINATION

A 35 years old lactating female with a known case of RHD presented to the emergency department of our hospital with chief complaints of inability to speak, right‐sided hemiparesis and chest pain for 6 h. Her inability to speak developed suddenly 6 h back, which was not associated with numbness, facial droop, dysphagia, headache, or vision changes and was progressively worsening. She had no problem writing and understanding the examiner. Similarly, she also had right‐sided hemiparesis involving both the upper and lower limbs, which was sudden in onset, non‐progressive, with no numbness to pain and temperature, and severe enough to limit her day‐to‐day activity. Moreover, she also had chest pain which was sudden in onset, burning in character, with no radiation and was associated with a general malaise. The pain was continuous in nature, with no known exacerbating or relieving factors.

From her past history, we found that she has been a patient of RHD for the past 10 years. She has no past history of diabetes mellitus and hypertension. However, her attendant told us that she had presented to the out‐patient department (OPD) of a nearby clinic 9 days back with chief complaints of anxiety, insomnia, malaise, palpitation, hot flushing and headache for 1 week. In the clinic, her physical examinations and laboratory investigations were mostly within normal limits except for an elevated pulse rate of 105 bpm, suggesting tachycardia. She was prescribed propranolol, flupenthixol and melitracen, metoclopramide hydrochloride, clonazepam, and obeticholic acid. She was asked to return for a follow up in 7 days, but did not comply with the follow‐up visit.

She reported the regular use of benzathine penicillin G intramuscular injection every other month since being diagnosed with RHD but had stopped using it in last 1 year. She also gives a history of five family members living in two‐bedroom flat, suggesting overcrowded living conditions. She has no significant personal or family history. She also gives no significant travel history in the last few months.

On physical examination of the patient, she was conscious, cooperative and well‐oriented to time, place and person. Her pulse was feeble and irregular, the blood pressure was elevated to 140/100 mmHg and she was afebrile. On head‐to‐toe examination, anemia and edema were absent. Also, the jugular venous pressure was within normal limits.

On cardiovascular examination, the inspection and palpation findings were normal. However, on auscultation, a murmur was detected over the mitral area.

On respiratory examination, the findings were unremarkable and the lung field was clear.

During the neurological examination, the assessment of motor function showed that the patient had fair muscle power with active movement present against gravity but not against resistance, which corresponds to Medical Research Council (MRC) Grade 3. On reflex examination, the extensor plantar response was present on the right side but absent on the left side. In addition to the motor and reflex examination, the other neurological examination findings including sensory function and higher mental functions were normal. On cranial nerve examination, all the findings were within normal limits. There was presence of gag reflex and uvula (pendulous veil) was situated towards the midline without deviation which suggests absence of dysphagia. On examination of the cerebellar functions, there was normal gait with no ataxia, tremor and dysdiadochokinesis. However, there was inability to speak confirming the presence of dysarthria.

## 3 METHODS (DIFFERENTIAL DIAGNOSIS, INVESTIGATIONS, AND TREATMENT)

**FIGURE 1:**
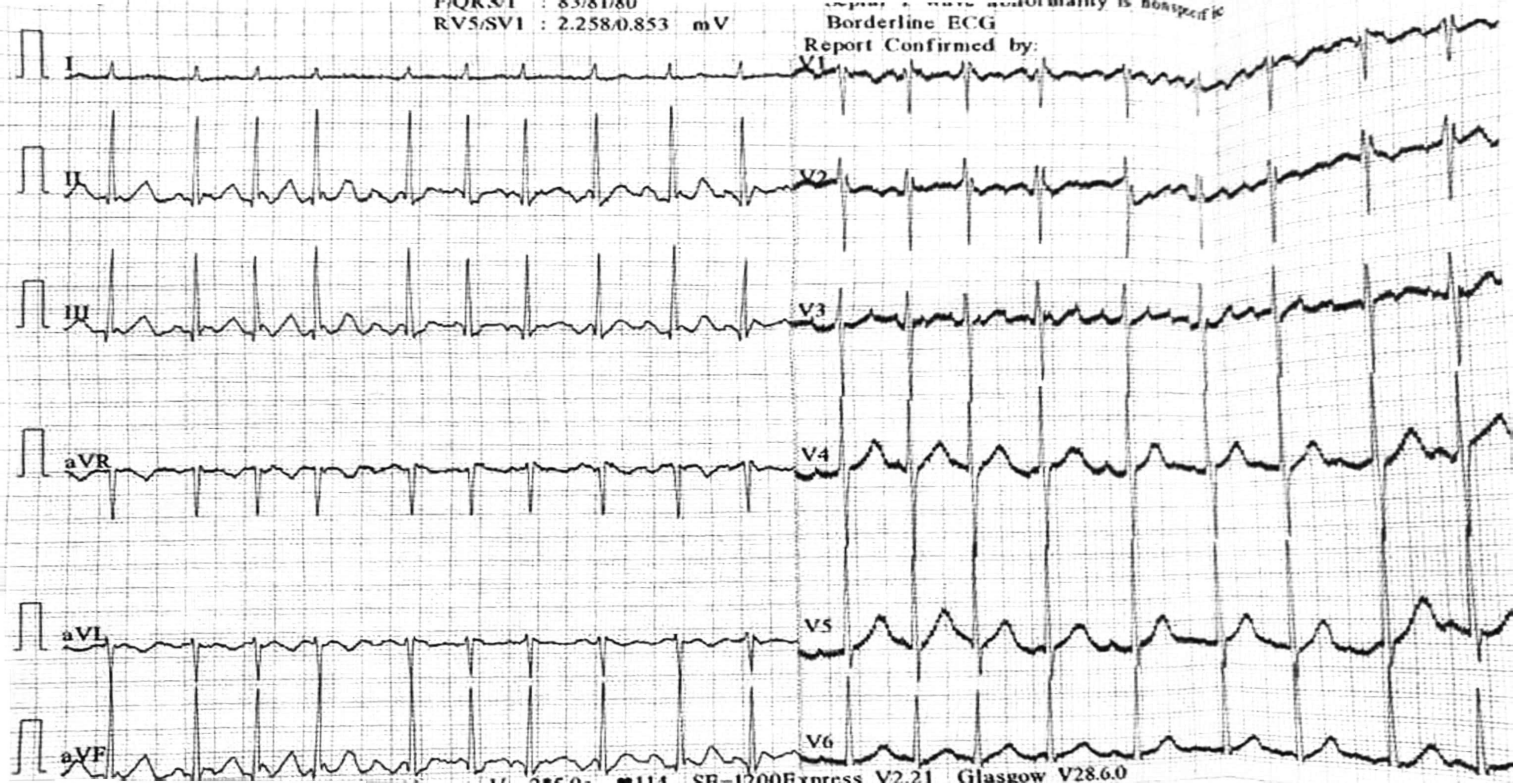
ECG showing irregularly irregular rhythm and narrow QRS complex.

As a result of the clinical findings, the doctor sent for complete blood count (CBC), which was within normal limits and electrocardiogram (ECG), which showed irregularly irregular rhythm and narrow QRS complex, which is suggestive of atrial fibrillation (Figure 1).

The patient was then immediately admitted to the critical care unit (CCU) for continuous assessment and monitoring.

Then, echocardiography and a computerized tomography (CT) scan were performed. The echocardiography findings were consistent with chronic rheumatic heart disease (CRHD). There was also moderate mitral stenosis (MS) and mitral regurgitation (MR), dilated left atrium (LA) but good left ventricle (LV) systolic function with no regional wall motion abnormality (RWMA). As a result, the atrial fibrillation detected on ECG can be attributed to RHD with significant involvement of the mitral valve. CT scan findings showed subtle ischemic changes in the right basal ganglia.

**FIGURE 2:**
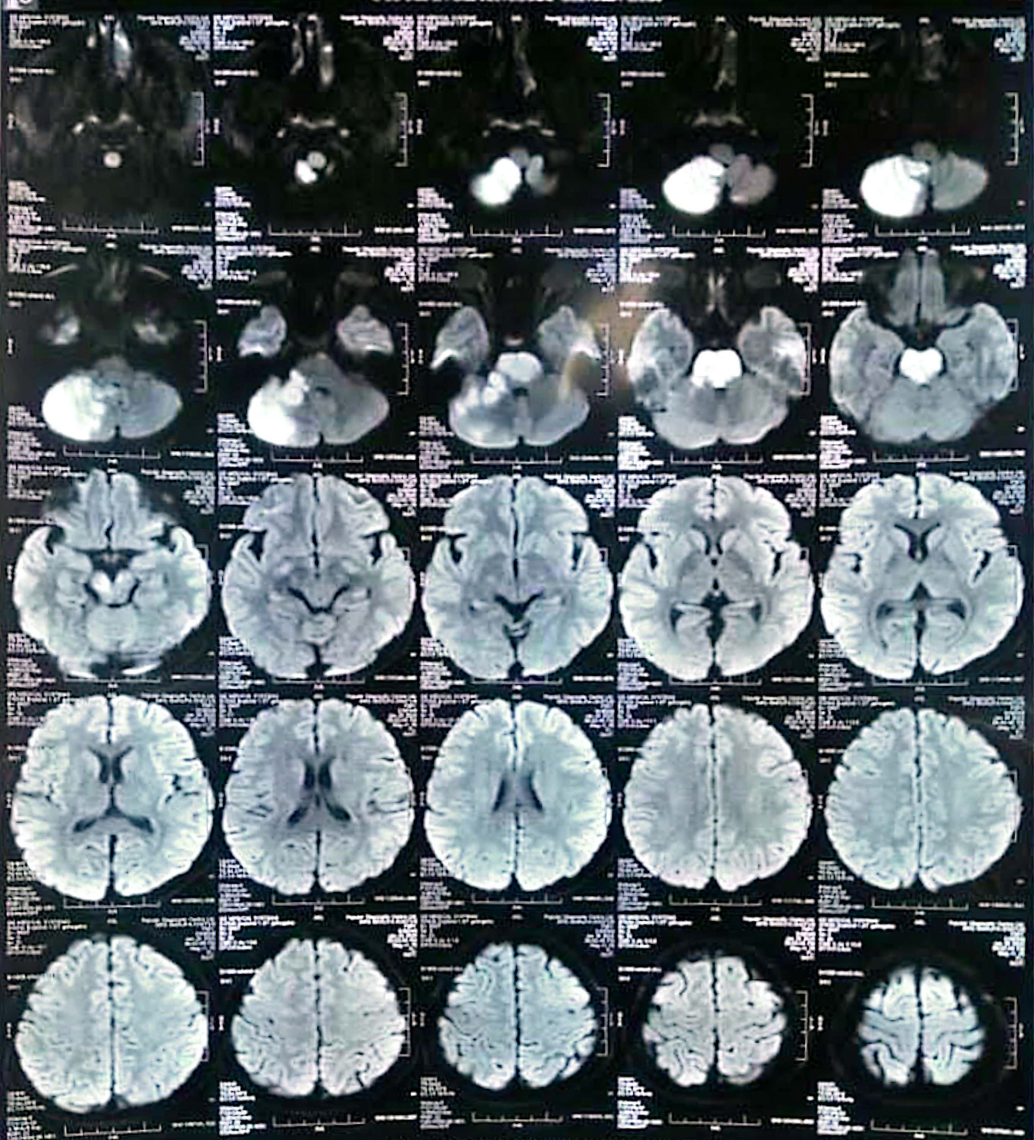
MRI brain showing acute infarct in right cerebellar hemisphere and pons.

Magnetic resonance imaging (MRI) of the brain with diffusion weighted imaging (DWI) was recommended, the results of which showed acute infarct in the right cerebellar hemisphere (in the PICA territory) and pons (Figure 2).

Following the radiological evaluation, the patient was diagnosed with LMS.

The patient was advised to maintain complete bed rest and receive nasogastric feeding. She was also prescribed medications which included nebulization with salbutamol, Ecosprin 75 mg, Penicillin V 250 mg, Metoprolol 50 mg, Losartan 50 mg, injectable Ceftriaxone, Vinocetine 5 mg, Piracetam 500 mg, Warfarin 5 mg, injectable Hydroxyprogesterone 25 mg as needed, injectable Prochlorperazine, Citicoline 500 mg and Dexamethasone 5 mg injection. The patient's treatment plan also included physiotherapy.

## 4 RESULTS (OUTCOME AND FOLLOW‐UP)

Despite management by medication and physiotherapy, the patient did not show improvement in symptoms and was referred to higher centers after 10 days of hospital stay.

## 5 DISCUSSION

RHD develops as a complication of rheumatic fever, an inflammatory condition triggered by a streptococcal infection.^5^ RHD can significantly damage the heart valves, especially the mitral valve, leading to a condition known as atrial fibrillation (AF), which is characterized by irregular and swift beating of the atria. The complication of AF is that it increases the risk of blood clot formation. When such clots travel to the PICA or VA, they occlude the blood flow, leading to an infarction in the medulla oblongata. This sequence of events leads to LMS.^2^, ^3^ Notably, this case is a rare instance of LMS arising from AF caused by RHD, which makes it a unique case report in the medical literature.

Acute Rheumatic fever (ARF) is caused by the body's autoimmune response to a throat infection caused by Streptococcus pyogenes, a group A Streptococcus bacterium (GAS).^6^ The primary prevention for GAS pharyngitis is intramuscular benzathine penicillin G. Secondary prevention involves continuous antibiotic chemoprophylaxis to prevent RF recurrences and reduce its risk of progression to RHD.^7^ Moreover, the treatment for AF in RHD ranges from rhythm/rate control medications to valve surgeries. Given the risks of stroke, the use of anticoagulation medications is recommended. However, anticoagulants are frequently under‐prescribed, potentially due to fear of bleeding or inadequate access to proper monitoring, particularly in remote and rural areas.^8^

RHD refers to the autoimmune damage to the heart caused by single or multiple episodes of ARF. This results in inflammation and progressive fibrosis of the heart valves, resulting in either or both valvular stenosis and regurgitation, typically over 2–3 decades.^6^ RHD is a major risk factor to the development of atrial fibrillation, especially when it involves the mitral valve.^9^

Before discussing LMS, it is important to discuss the posterior circulation, which comprises two vertebral arteries, a basilar artery, two posterior cerebral arteries and their branches, responsible for approximately 20% of all ischemic strokes. Among the arteries in the posterior circulation, the PICA plays a major role in the blood supply of the cerebellum. PICA originates from a VA. The PICA territory infarcts are the most common type of cerebellar ischemic stroke, accounting for 40%. The dorsolateral medulla is supplied by the medullary branches and is responsible for the LMS. This area is the most frequently involved ischemic region of the PICA infarct. However, the majority of these PICA territory infarcts, including LMS can be attributed to the VA from which PICA arises (67%), as opposed to PICA itself (10%).^10^

A study by Kumral et al. showed that extracranial large‐artery disease, cardio embolism and in situ branch disease were the major causes of PICA infarcts.^11^

A study by Baran et al. suggested that medullary infarcts were often associated with cerebellar infarcts, mesencephalic infarcts often occurred alongside posterior cerebral artery infarcts and pons infarcts were linked to infarcts in the anterior circulation and multiple infarcts.^12^ Similarly, in our case, the patient had also presented with a coinciding pons and cerebellar infarct, both of which fall under PICA territory.

In a study by Toyoda et al., embolic heart disease was identified as the cause in 30% of the 73 cases of acute pontine infarction.^13^ Although not the most common etiology, cardio embolism remains a significant cause of isolated pontine infarct, cerebellar infarct, or the co‐occurrence of both pontine and cerebellar infarct. Thus, the embolus due to AF resulting from RHD can lead to LMS.

LMS usually presents with classical triad of symptoms which include Horner's syndrome, ataxia on the same side and reduced sensitivity to pain and temperature on the same side. It can also presents with nausea, vomiting as well as cerebellar manifestations.^14^ However, it is possible that LMS presents with atypical symptoms as well. In our case report, the patient presented with dysarthria and right sided hemiparesis. This is a deviation from the expected clinical presentation of LMS. This prompted further investigation to understand the underlying pathology. The clinical findings were confirmed by radiological findings which revealed acute infarction in right cerebellar hemisphere within the PICA territory and the pons.

The right sided hemiparesis can due to caudal extension of the infract affecting the corticospinal fibers post decussation. However, the recent findings suggest that it may be due to involvement of medullary penetrating arteries, branch of VA which supply the pyramidal fibers after decussation. The presence of extensor plantar response suggests that the pyramidal tract was involved.^15^ Dysarthria is likely due to cerebellar or brainstem involvement. It can be due to cerebellar infarct, brainstem infarct or both.^16^

This case presents a rare LMS associated with RHD in a young female. The patient arrived to the hospital with a clinical presentation of stroke. Still, the clinical triad of a LMS like Horner's syndrome, ipsilateral ataxia and hypalgesia and thermoanesthesia of the ipsilateral face was absent.^14^ The confirmatory diagnosis of LMS was based on radiological evidence. In countries with low socioeconomic status, RHD still remains prevalent, often undiagnosed and picked up on echocardiography when presented with cardiac‐related complications. Therefore, hygiene‐related awareness and vigilance regarding GAS pharyngitis/rheumatic fever in early stages using Jones criteria, anti‐streptolysin O (ASO) titer, which constitute primordial and primary prevention, as well as complete secondary prophylaxis remain vital in diminishing adverse cardiac outcomes. In any case among all age groups with known heart disease, strictly treating the arrhythmias to avoid the debilitating outcome “stroke” is necessary. Further, regular surveillance for cardiac changes in known cases and appropriate management of arrhythmias, including the use of anticoagulants where warranted, will serve to avoid the debilitating outcome “stroke” leading to LMS.

In conclusion, the association of RHD, leading to atrial fibrillation and resulting in LMS, is rarely reported in case studies. Clinicians should consider the potential of LMS as a complication of RHD and atrial fibrillation, conduct effective surveillance of cardiac changes, strictly manage arrhythmias and introduce prophylactic stroke medications where monitoring is possible. Further research and studies on this topic are of utmost importance.

## AUTHOR CONTRIBUTIONS

**Sajina Shrestha:** Conceptualization; writing – original draft; writing – review and editing. **Suda Maharjan:** Conceptualization; writing – original draft; writing – review and editing. **Bandana Ghimire:** Conceptualization; writing – original draft; writing – review and editing. **Nischal Mainali:** Conceptualization; writing – original draft; writing – review and editing. **Kriti Gurung:** Conceptualization; writing – original draft; writing – review and editing. **Hritik Raj Yadav:** Conceptualization; writing – original draft; writing – review and editing. **Kriti Bhandari:** Conceptualization; writing – original draft; writing – review and editing. **Shumneva Shrestha:** Conceptualization; writing – original draft; writing – review and editing. **Anupam Halder:** Conceptualization; writing – original draft; writing – review and editing. **Kripa Rajak:** Conceptualization; writing – original draft; writing – review and editing. **Vikash Jaiswal:** Supervision.

## FUNDING INFORMATION

This research did not receive any specific grant from funding agencies in the public, commercial, or not‐for‐profit sectors.

## CONFLICT OF INTEREST STATEMENT

The authors declare that there is no conflict of interest.

## ETHICS STATEMENT

Not applicable.

## CONSENT

Written informed consent was obtained from the patient for publication of this case report and any accompanying images. A copy of the written consent will be available for review if asked by the editor‐in‐chief of this journal.

## Data Availability

Data sharing is not applicable to this article as no new data were created or analyzed in this study.
